# Seismicity Pattern Changes Prior to the 2008 Ms7.3 Yutian Earthquake

**DOI:** 10.3390/e21020118

**Published:** 2019-01-28

**Authors:** Qinghua Huang

**Affiliations:** Department of Geophysics, School of Earth and Space Sciences, Peking University, Beijing 100871, China; huangq@pku.edu.cn

**Keywords:** seismicity, seismic quiescence, Region-Time-Length (RTL) method, Yutian earthquake

## Abstract

Seismicity pattern changes that are associated with strong earthquakes are an interesting topic with potential applications for natural hazard mitigation. As a retrospective case study of the Ms7.3 Yutian earthquake, which was an inland normal faulting event that occurred on 21 March 2008, the Region-Time-Length (RTL) method is applied to the seismological data of the China Earthquake Administration (CEA) to analyze the features of the seismicity pattern changes before the Yutian earthquake. The temporal variations of the RTL parameters of the earthquake epicenter showed that a quiescence anomaly of seismicity appeared in 2005. The Yutian main shock did not occur immediately after the local seismicity recovered to the background level, but with a time delay of about two years. The spatial variations of seismic quiescence indicated that an anomalous zone of seismic quiescence appeared near the Yutian epicentral region in 2005. This result is consistent with that obtained from the temporal changes of seismicity. The above spatio-temporal seismicity changes prior to the inland normal faulting Yutian earthquake showed similar features to those reported for some past strong earthquakes with inland strike faulting or thrust faulting. This study may provide useful information for understanding the seismogenic evolution of strong earthquakes.

## 1. Introduction

**Figure 1:**
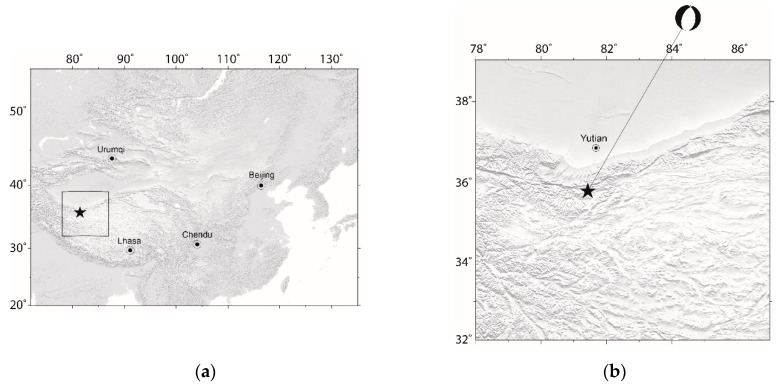
The 2008 Ms7.3 Yutian earthquake and the investigated region in this study. The star represents the epicenter of the Ms7.3 Yutian earthquake. (**a**) Map including the investigated region (marked by the rectangular) and some main cities in China; (**b**) The investigated region in this study. The focal mechanism of the Yutian earthquake is from [3].

An Ms7.3 earthquake (Figure 1) struck Yutian County, Xinjiang Uygur Autonomous Region, China on 21 March 2008 [1,2]. The moment magnitude of the Yutian earthquake was M_w_ 7.1 [3]. Instead of the M_w_, which is a well adopted magnitude for earthquakes, the Ms unit is adopted in this study because it is the officially reported magnitude of earthquakes by China Earthquake Administration (CEA). The epicenter locations and rupture models that have been reported by several earthquake agencies are very different from each other [1]. To have a better understanding about the seismogenic structure and rupture process of the Ms7.3 Yutian earthquake, Institute of Geology, CEA and Earthquake Administration of Xinjiang Uygur Autonomous Region made a joint scientific investigation on the Ms7.3 Yutian earthquake and the Ashikule Volcanoes in May 2011 [3]. The related results indicated that the Ms7.3 Yutian earthquake is an inland normal faulting event that combines with the partial left-lateral faulting component [1,3] (also see Figure 1b for the focal mechanism of the Yutian earthquake).

There are some retrospective studies on the 2008 Ms7.3 Yutian earthquake, such as seismogenic structure [2,3], seismicity changes [2,4], gravity changes [5], infrared anomalies [6], co-seismic deformation [7], and so on. The related results have provided some useful information for understanding the seismogenic structure and rupture process of the Yutian earthquake. Among the above reported changes possibly associated with earthquakes, seismicity changes may provide some useful information for intermediate-term forecasts of earthquakes [8], as even earthquake forecasting has been a controversial issue for a long time [8,9,10,11]. It is noteworthy that recently, analyses of seismicity based on information entropy (e.g., [12]), Tsallis entropy (e.g., [13]), and natural time entropy (e.g., [14]) have provided interesting results towards intermediate-term and short-term forecasting.

As one of the seismicity analysis methods, the Region-Time-Length (RTL) method, which considers the contributions of the epicenter, time, and magnitude of earthquakes, showed positive results for some case studies in various tectonic regions [8,15,16,17,18,19,20]. Different from the previous case studies for earthquakes with strike faulting or thrust faulting, the 2008 Ms7.3 Yutian earthquake is an inland normal faulting event. Is there any seismicity change prior to the Yutian earthquake? Are the characteristics of seismicity changes of the inland normal faulting event similar to or different from those of the previously reported earthquakes with strike faulting or thrust faulting? A study on these questions should be an interesting topic for those who want to understand the seismogenic physics of strong earthquakes. Thus, the RTL method is adopted in this study to investigate seismicity pattern changes before the Yutian earthquake. The similarities/differences in seismicity changes between the Yutian earthquake and some case studies reported previously, are also discussed. This study may provide some useful information for understanding the seismogenic physics of strong earthquakes, and it can strengthen the potential application of spatio-temporal seismicity in seismic risk assessment.

## 2. Data and Methods

The seismological data of earthquake catalog are from CEA. Because the CEA earthquake catalog has been well-documented since 1 January 1970, the catalog with a time window from 1 January 1970 to 21 March 2008 (the occurrence time of the investigated Ms7.3 Yutian earthquake) was chosen as the basic data in this study.

The RTL method is adopted in this study to investigate the seismicity changes of the Yutian earthquake. The details of the RTL method can be referred to some previous publications [15,17,18]. The basic idea of the RTL method is that each prior event has some weighted influence on the main event under investigation. The RTL parameter is defined mathematically as the product of the following three normalized functions that are respectively related to the epicenter, the occurrence time, and the magnitude of an event [18]:

(1) $$\begin{array}{l} R(x,y,z,t)=\left[\sum_{i}\exp(-\frac{r_{i}}{r_{0}})I(r_{i}\leq 2r_{0})I(t-t_{i}\leq 2t_{0})I(d_{i}\leq d_{0})I(M_{i}\geq M_{\min})\right]-R_{bk}(x,y,z,t)\\ T(x,y,z,t)=\left[\sum_{i}\exp(-\frac{t-t_{i}}{t_{0}})I(r_{i}\leq 2r_{0})I(t-t_{i}\leq 2t_{0})I(d_{i}\leq d_{0})I(M_{i}\geq M_{\min})\right]-T_{bk}(x,y,z,t)\\ L(x,y,z,t)=\left[\sum_{i}\left(\frac{l_{i}}{r_{i}}\right)I(r_{i}\leq 2r_{0})I(t-t_{i}\leq 2t_{0})I(d_{i}\leq d_{0})I(M_{i}\geq M_{\min})\right]-L_{bk}(x,y,z,t) \end{array}$$

where *R*(*x*, *y*, *z*, *t*), *T*(*x*, *y*, *z*, *t*) and *L*(*x*, *y*, *z*, *t*) are functions of epicentral distance, occurrence time, and rupture length, respectively; *I*(Ω) is the following logical function:

(2) $$I(\Omega )=\left\{ \begin{array}{ccc} 1, & & \Omega \;is\;true\\ 0, & & otherwise \end{array}\right.;$$

*l_i_* is the rupture length related to the magnitude *M*_i_ of the *i*th event; *t_i_* is the occurrence time of the *i*th event; *r_i_* is the epicentral distance from the investigated position (*x*, *y*, *z*) to the epicenter of the *i*th event; *r*_0_ and *t*_0_ are the characteristic distance and time-span, respectively; *d_i_* is the focal depth of the *i*th event; *d*_0_ is the cut-off depth; *M*_min_ is the cut-off magnitude for a complete earthquake catalog; *R*_bk_(*x*, *y*, *z*, *t*), *T*_bk_(*x*, *y*, *z*, *t*) and *L*_bk_(*x*, *y*, *z*, *t*) are the trends (background values) of *R*(*x*, *y*, *z*, *t*), *T*(*x*, *y*, *z*, *t*) and *L*(*x*, *y*, *z*, *t*), respectively.

The above three functions of *R*(*x*, *y*, *z*, *t*), *T*(*x*, *y*, *z*, *t*) and *L*(*x*, *y*, *z*, *t*) in Equation (1) are further normalized respectively by their standard deviations, *σ_R_*, *σ_T_*, and *σ_L_*. The RTL parameter is defined as the product of these normalized dimensionless functions. Hence, the RTL parameter can describe the seismicity changes, compared to the background level. A negative RTL parameter represents a decrease in seismicity, and a positive one shows an increased seismicity [18].

Besides the temporal variation of seismicity as revealed by the RTL parameter, the Q parameter was developed to quantify the quiescence anomaly of seismicity at a certain position [21]. The parameter *Q*(*x*, *y*, *z*, *t*_A_, *t*_B_) at an investigated position (*x*, *y*, *z*) and a time window [*t*_A_, *t*_B_] is defined as an average of the RTL values over the investigated time window [*t*_A_, *t*_B_]:

(3) $$Q\left(x,y,z,t_{A},t_{B}\right)=\frac{1}{m}\sum_{j=1}^{m}RTL\left(x,y,z,t_{j}\right)$$

where *t_j_* is the time in the time window [*t*_A_, *t*_B_] for calculating the *Q* parameter; *RTL*(*x*, *y*, *z*, *t_j_*) is the RTL parameter at the investigated position (*x*, *y*, *z*), calculating from the start time of a complete earthquake catalog to time *t_j_*; *m* is the total number of RTL parameters that are available in the time window [*t*_A_, *t*_B_]. The *Q* parameter defined by Equation (3) can describe quantitatively the spatial map of quiescence anomaly of seismicity in the time window [*t*_A_, *t*_B_] with respect to the background seismicity from the start time of a complete earthquake catalog to the ending time *t*_B_. Some case studies indicated that the *Q* parameter is effective in quantifying the spatial map of quiescence anomaly of seismicity [18,19,22].

## 3. Results

### 3.1. Pre-Processing of Earthquake Catalog Data

**Figure 2:**
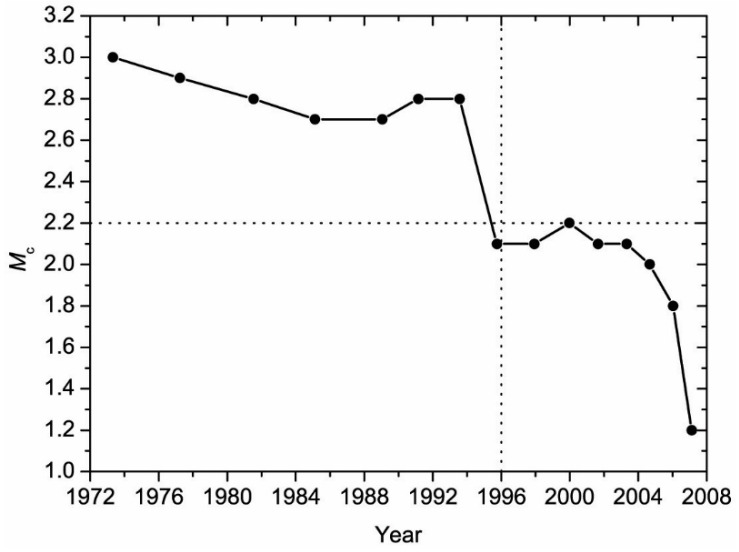
Temporal variation of the completeness magnitude (M_c_) of the China Earthquake Administration (CEA) earthquake catalog in Yutian area. The vertical dashed line indicates the starting time of the catalog with a complete magnitude (M_c_) of 2.2.

It is well-known that artificial seismicity changes may arise from the changes of the seismic network, e.g., station distribution, upgrades of seismic instruments and data management software, etc. [18,23]. To minimize the possible artificial effect on seismicity changes, it is important to have some pre-processing of the seismological data of earthquake catalog before the application of the RTL method. The data pre-processing mainly includes aftershock declustering and completeness analysis [18]. The aftershocks of the CEA earthquake catalog were declustered using the approach given in [24]. The completeness of the CEA earthquake catalog was evaluated based on the power-law of frequency–magnitude [25]. Figure 2 shows the temporal change of the completeness magnitude of the CEA earthquake catalog in Yutian area. The result indicates that the completeness magnitude decreases with time (Figure 2), i.e., the detectability of the earthquakes increases with time. As shown in Figure 2, the CEA earthquake catalog in Yutian area is complete for earthquake with magnitude M ≥ 2.2 after 1 January 1996 (marked by the vertical dashed line). Therefore, the CEA earthquake catalog from 1 January 1996 to 21 March 2008 with a completeness magnitude (M_c_) of 2.2 is chosen in this study, which can satisfy both the completeness of the earthquake catalog, and enough number of events for seismicity analysis.

### 3.2. Temporal Variation of Seismicity

**Figure 3:**
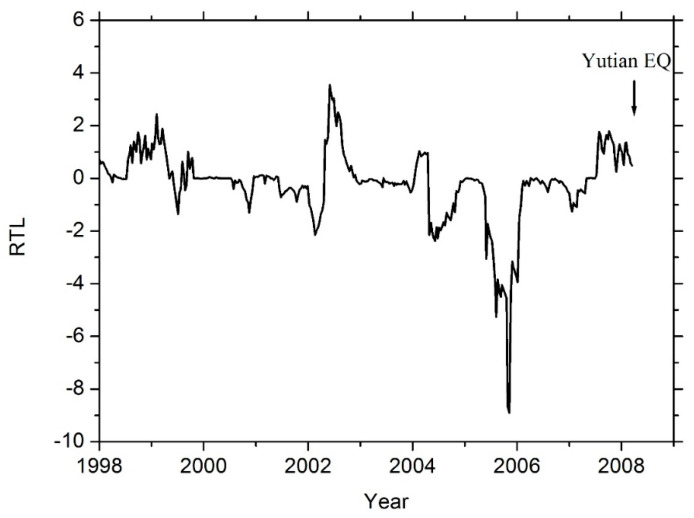
Temporal variation of the calculated Region-Time-Length (RTL) parameter in the epicentral area of the 2008 Ms7.3 Yutian earthquake. The vertical arrow indicates the occurrence time of the Yutian earthquake. A clear quiescence appeared in 2005.

Applying the RTL method to the above declustered and complete earthquake catalog in Yutian area, one can obtain the seismicity changes in the same area. Due to the selection of the characteristic time span (*t*_0_) in Equation (1), the calculated RTL parameters will be available after a time window of 2*t*_0_ from the starting investigated time of the earthquake catalog [18]. In this study, the characteristic time span is adopted as *t*_0_ = 1 year, and the start of the investigated time of earthquake catalog is 1 January 1996 (as shown in Section 3.1). Hence, the available time window of the calculated RTL parameters is from 1 January 1998 to 21 March 2008 (as shown in Figure 3). Figure 3 gives the temporal change of the seismicity, which is revealed by the calculated RTL parameters, in the epicentral area of the 2008 Ms7.3 Yutian earthquake. The result shows that a quiescence anomaly of seismicity began in 2004, and developed in 2005 in the Yutian area. The seismicity recovered to background levels in 2006. The Ms7.3 Yutian earthquake did not occur immediately after the seismicity recovered to the background level. Instead, it occurred with a time delay of about two years, as indicated by the vertical arrow in Figure 3.

### 3.3. Spatial Distribution of Seismicity

**Figure 4:**
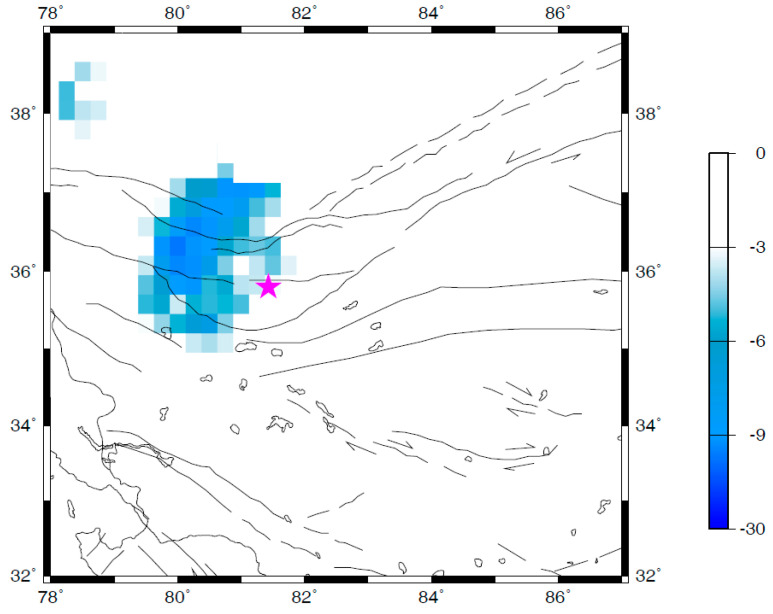
Spatial distribution of the seismic quiescence quantified by the *Q* parameter during July–December 2005. The star represents the epicenter of the Yutian earthquake. The lines represent the main faults. The arrows indicate the strike directions of faults.

Besides the temporal change of seismicity, the spatial distribution of seismicity can strengthen the reliability of the correlation between the revealed seismicity changes and the target earthquake. The *Q* parameter, which is defined by Equation (3), was adopted to quantify and investigate the spatial variations of the quiescence anomaly of seismicity associated with the Ms7.3 Yutian earthquake. The interval of the time window [*t*_A_, *t*_B_] was chosen as six months in this study, i.e., *t*_B_ − *t*_A_ = 6 months. Although the selection of this interval was somehow empirical [18,21], the key concern was that the target time scale for seismicity changes in this study was in order of about one year. A sliding interval of six months in a time window should be enough to reveal the temporal evolution of the Q-map (the map of quiescence anomaly of seismicity). Therefore, one can obtain the Q-map for each time interval of six months (i.e., the time window [*t*_A_, *t*_B_]) with respect to the background seismicity from the start time of the adopted complete earthquake catalog (i.e., 1 January 1996 in this study) to the ending time *t*_B_. The temporal changes of the Q-map can be obtained by sliding the calculated ending time of the earthquake catalog (i.e., *t*_B_ in this study). The temporal change of seismicity in Yutian area showed that the most significant quiescence appeared at around late 2005 (Figure 3). As an example, Figure 4 shows the spatial map of the quiescence anomaly of seismicity in the investigated region for a time window [*t*_A_, *t*_B_] of July–December 2005. An anomalous zone of seismic quiescence appeared near the epicentral region of the Yutian earthquake (the epicenter is marked by a star in Figure 4). The linear dimension of the anomalous quiescence zone was about 200 km (Figure 4).

## 4. Discussion

Both the temporal change (Figure 3) and the spatial map (Figure 4) of seismicity in Yutian area showed that a seismic quiescence appeared near the epicenter about 2.5 years before the Ms7.3 Yutian earthquake. In fact, the 2008 Ms7.3 Yutian earthquake is the only main event with Ms ≥ 6.5 in the investigated region (as shown in Figure 4), and within the available time window of the calculated RTL parameters (1 January 1998–21 March 2008, as shown in Figure 3).

**Figure 5:**
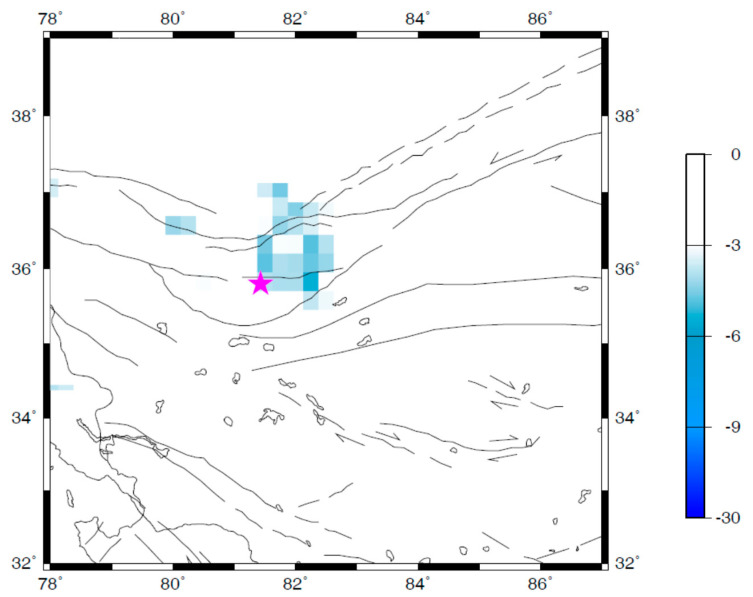
Spatial distribution of the seismic quiescence as quantified by the *Q* parameter during July–December 2004. The star represents the epicenter of the Yutian earthquake. The lines represent the main faults. The arrows indicate the strike directions of faults.

**Figure 6:**
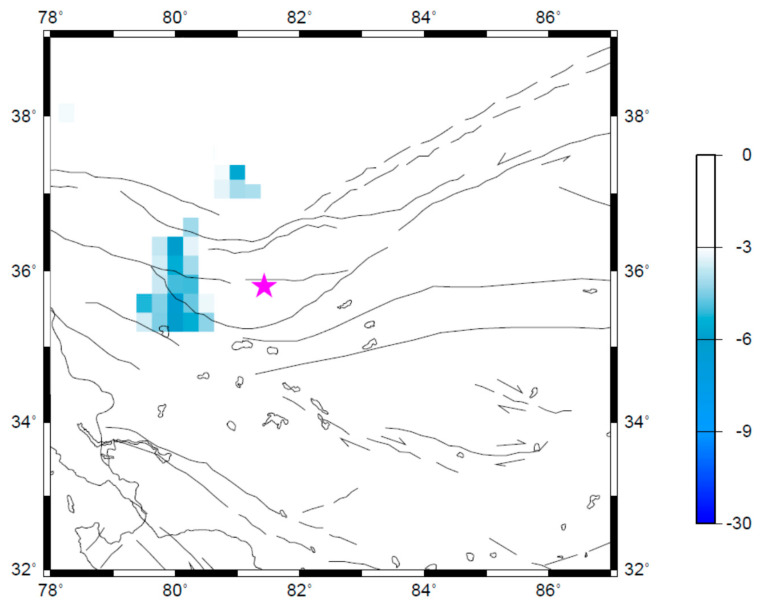
Spatial distribution of the seismic quiescence as quantified by the *Q* parameter during January–June 2006. The star represents the epicenter of the Yutian earthquake. The lines represent the main faults. The arrows indicate the strike directions of faults.

To further understand the spatio-temporal characteristics of the seismicity changes, the Q-map in the investigated region (shown in Figure 1) is calculated for various time window [*t*_A_, *t*_B_]. The results showed that almost no any quiescence anomaly is detected in the investigated region before late 2003. Seismic quiescence tends to appear near the epicenter in 2004 (Figure 5), and develop until late 2005 (Figure 4). Then, the anomalous zone of seismic quiescence becomes smaller in early 2006 (Figure 6). The quiescence anomaly of seismicity disappears after late 2006. The above temporal evolution of the spatial map of quiescence anomaly of seismicity indicates the plausible correlation with the development of the Ms7.3 Yutian earthquake, at least in the spatio-temporal domain.

The temporal evolution of the spatial map of quiescence anomaly of the Ms7.3 Yutian earthquake revealed in this study shows some similar characteristics to previously reported case studies in various tectonic regions [8,18,19,20,21,22]. A seismic quiescence quantified by the RTL parameter appears a few years before the Yutian earthquake. The Q-map reveals an anomalous zone of seismic quiescence near the epicenter. The length of the anomalous zone of seismic quiescence is about 200 km, several times the rupture length of the Ms7.3 Yutian earthquake (Figure 4). The Yutian earthquake occurred near the boundary of the anomalous zone of seismic quiescence (Figure 4). The similarity in the spatio-temporal characteristics of seismicity for earthquakes with different tectonics and focal mechanisms may reflect the natural evolution of seismogenic process.

Generally, an earthquake is most likely to occur after the relevant source region has passed through seismic quiescence, and is in the stage of coming back to background seismicity. Close investigation of both the temporal variation (Figure 3) and the spatial distribution (Figure 4) of seismicity would provide useful information for seismic risk assessments. However, the Ms7.3 Yutian earthquake did not occur immediately after the seismic quiescence stage in the source region. Instead, it occurred with a time delay of about two years after the seismicity recovered to background levels in the source region. Similar features of time delay were reported previously for the M_w_7.4 Izumit earthquake in Turkey [21]. The existence of a time delay after the seismic quiescence makes it difficult to determine the occurrence time of a future event with the accuracy of short-term forecasting.

## 5. Conclusions

The seismicity changes of the 2008 Ms7.3 Yutian earthquake were investigated by applying the RTL method to CEA seismological data. The temporal variation of seismicity in Yutian area showed a significant quiescence anomaly in 2005, about 2.5 years before the mainshock. The Yutian earthquake did not occur immediately after the seismicity recovered to the background level, but with a time delay of about two years. The Q-map (a spatial map of quiescence anomaly) indicated that an anomalous zone of seismic quiescence appeared in 2005 near the Yutian epicentral area, consistent with the features revealed by the temporal variation of the RTL parameters. The size of the anomalous quiescence zone is about 200 km, several times of the rupture length of the main shock. The retrospective case study on the Yutian earthquake showed a combination of a temporal change and a spatial map of seismicity would provide useful information for seismic risk assessments.
